# Nonadiabatic Dynamics Simulation Predict Intersystem Crossing in Nitroaromatic Molecules on a Picosecond Time Scale

**DOI:** 10.1002/cptc.201900108

**Published:** 2019-06-13

**Authors:** J. Patrick Zobel, Leticia González

**Affiliations:** ^1^ Division of Theoretical Chemistry, Kemicentrum Lund University P.O. Box 124 SE-221 00 Lund Sweden; ^2^ Institute of Theoretical Chemistry University of Vienna Währinger Straße 17 A-1090 Vienna Austria

**Keywords:** ab initio calculations, computational photochemistry, intersystem crossing, nitroaromatic molecules, nonadiabatic dynamics, photophysics

## Abstract

Previous time‐resolved spectroscopic experiments and static quantum‐chemical calculations attributed nitronaphthalene derivatives one of the fastest time scales for intersystem crossing within organic molecules, reaching the 100 fs mark. Nonadiabatic dynamics simulations on three nitronaphthalene derivatives challenge this view, showing that the experimentally observed ∼100 fs process corresponds to internal conversion in the singlet manifolds. Intersystem crossing, instead, takes place on a longer time scale of ∼1 ps. The dynamics simulations further reveal that the spin transitions occur via two distinct pathways with different contribution for the three systems, which are determined by electronic factors and the torsion of the nitro group. This study, therefore, indicates that the existence of sub‐picosecond intersystem crossing in other nitroaromatic molecules should be questioned.

## Graphical abstract

**SHARC Bites Again**: Nonadiabatic dynamics simulations reveal the photodeactivation mechanism of three nitronaphthalene derivatives. A subpicosecond process previously attributed to intersystem crossing is identified as internal conversion within the singlet states. An easy prey for the SHARC.

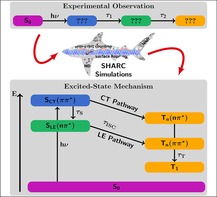

## 1 Introduction

Intersystem crossing (ISC) is a fundamental process to selectively convert light into other forms of energy in molecules, e. g., by charge separation. It connects electronic states of different spin multiplicity, and, in a simple picture, its efficiency depends on the energetic difference between the electronic states as well as the strength of their non‐adiabatic coupling and spin‐orbit coupling (SOC). When the SOCs are large, ISC transitions are expected to occur very effectively and can take place on an ultrafast time scale up to femtoseconds. This is often found in metal complexes, where the heavy (metal) atoms provides large SOCs.^1^ In contrast, ISC is typically several orders of magnitudes slower in organic molecules. This is rationalized by the smaller SOCs, which should make ISC transitions less efficient in comparison.

Despite this general rule of thumb, a number of organic molecules have been reported to display ultrafast ISC on a sub‐picosecond time scale after photoexcitation, despite possessing small SOCs. Of these molecules, a large part belong to the class of nitro polycyclic aromatic hydrocarbons (NPAHs)^2^, ^3^, ^4^, ^5^, ^6^, ^7^, ^8^, ^9^, ^10^, ^11^, ^12^, ^13^, ^14^ or the closely related nitrobenzene derivatives.^15^, ^16^, ^17^, ^18^, ^19^, ^20^, ^21^, ^22^, ^23^, ^24^, ^25^, ^26^, ^27^ The assumptions about the processes occurring after excitation and the existence of ultrafast ISC are derived mainly from the interpretation of time‐resolved spectroscopic experiments. A small number of studies reported calculations on electronic excited states and potential energy surfaces along selected nuclear coordinates^28^, ^29^, ^30^, ^31^, ^32^ to aid rationalizing the experimental observations of these nitroaromatic compounds. However, dynamical studies that can directly identify the electronic states playing a role during the time evolution of the system as well as the nuclear motion connecting these states, are much more scarce.

The first excited‐state dynamics simulations on nitroaromatic molecules were performed by Xu and co‐workers for different nitrophenoles.^33^, ^34^ They attributed ISC to occur within less than 25 fs, i. e., even faster than the scale of a (few) hundreds of femtoseconds suggested in previous experiments.^27^ Recently, we performed an excited‐state dynamics study on a NPAH derivative, 2‐nitronaphthalene (2NN),^35^, ^36^ for which ISC was suggested to occur experimentally on a ∼100 fs time scale.^6^, ^7^ However, our simulations did not confirm this assumption. Instead, we attributed relaxation within the singlet manifold to the ∼100 fs process, which is then followed by ISC on a defiantly longer ∼1 ps time scale. Based on our simulation, a new deactivation mechanism for 2NN was proposed which, while differing from the one suggested previously,^2^, ^3^, ^4^, ^5^, ^6^, ^7^, ^8^ could still explain the time constants for electronic decay processes measured in the same experimentally studies. Intrigued by these controversial results, in this work we investigate the excited‐state dynamics of another two nitronaphthalene (NN) derivatives, 1‐nitronaphthalene (1NN) and 2‐methyl‐1‐nitronaphthalene (2M1NN), for which ISC has also been suggested to occur on a femtosecond time scale.

The rest of this work is organized as follows. After we introduce our methodology (section 2), we combine our new results with our previous studies on 2NN (section 3). We identify the common characteristics in the electronic states that enable ISC in NN derivatives as well as the factors responsible for the two different ISC pathways (section 3). We then review the previously established experimental and theoretical findings on the excited‐state dynamics of NN derivatives in more detail (section 4) to contrast these findings with our own results (section 5). Finally, we conclude our study and identify the open questions to be addressed in future studies (section 6).

## 2 Computational Details

The excited‐state dynamics of 2NN, 1NN, and 2M1NN were simulated in gas phase using the surface hopping including arbitrary couplings (SHARC) approach.^37^, ^38^, ^39^ Following our previous work on 2NN^40^ we employ density functional theory (DFT) and time‐dependent DFT (TDDFT) calculations at the PBE0^41^, ^42^/DZP^43^ level of theory using the ADF2016 program package^44^ and the SHARC/ADF interface.^45^ For 2NN, we did not perform new surface hopping simulations, but re‐use the data from our previous simulations^35^ and add new analyses.

### 2.1 Initial Conditions and Absorption Spectrum

Before simulating the excited‐state dynamics, a ground‐state geometry optimization was performed and normal modes and vibrational frequencies were calculated at the minimum‐energy geometry. Then, for each molecular system, 1000 initial conditions were sampled around the optimized geometry using a harmonic Wigner distribution at a temperature of T=300 K.^36^ For each geometry, the 10 lowest excited singlet and triplet states were calculated in the Tamm‐Dancoff approximation (TDA) using a Becke integration grid and a ZlmFit grid of normal quality.

**Figure 1:**
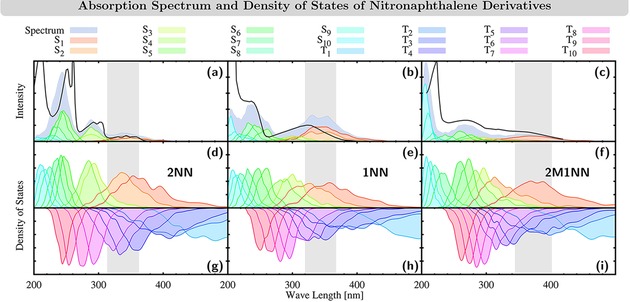
(a–c) Calculated gas‐phase absorption spectra of 2NN, 1NN, and 2M1NN, individual state contributions to the spectra, and experimental absorption spectra (black line) of 2NN and 1NN in n‐heptane^55^ as well as of 2M1NN (solvent not reported).^[56]^ Panels (d–f)/(g–i): Density‐of‐states of singlet/triplet states of 2NN, 1NN, and 2M1NN calculated at the PBE0/DZP level of theory performed for ensembles obtained from Wigner phase‐space ensembles at *T*=300 K. Other available absorption spectra of 2M1NN^7^ show a slow but steady rise in intensity towards smaller wave lengths. These spectra showed no discernible features until ca. 300 nm (cyclohexane) and 285 nm (acetonitrile), when the rise of intensity becomes larger, i. e., indicating the onset of the second absorption band. As this second band as well as a third band are clearly visible in the spectrum of unknown solvent,^56^ we included this spectrum instead in this Figure. In the spectrum of unknown solvent, the onset of the second absorption band is found at 300 nm, i. e., at the same wave length as that of 2M1NN in cyclohexane.^7^

Scalar relativistic effects were included in the zeroth‐order regular approximation (ZORA) and SOCs were calculated perturbatively as implemented in ADF2016.^44^ Using the geometries of the initial conditions, the absorption spectrum and the density of states (Figure 1) were simulated by Gaussian convoluting the oscillator‐weighted and unweighted stick spectra, respectively, using a FWHM of 0.1 eV.

### 2.2 Trajectories Set‐Up

For 1NN and 2M1NN, 75 trajectories were set‐up from the Wigner ensemble. For 2NN, we re‐use the data from the 105 trajectories propagated in our previous study.^35^

For each molecule, we chose initial conditions to setup the trajectories stochastically^46^ in a 0.5 eV window around the maximum of the first absorption band (see gray areas in Figure 1). Based on the density‐of‐states at the initial conditions, the following singlet and triplet states were included in the SHARC simulations: *S*_0_‐*S*_2_/*T*_1_‐*T*_6_ for 2NN, *S*_0_‐*S*_4_/*T*_1_‐*T*_6_ for 1NN, and *S*_0_‐*S*_4_/*T*_1_‐*T*_6_ for 2M1NN. For each molecule, two additional singlet and triplet states were calculated as inactive states.

### 2.3 SHARC Simulations

Each trajectory was propagated during 500 fs using a nuclear time step of 0.5 fs and an electronic time step of 0.02 fs within the local diabatization method.^47^ The 500 fs time window was chosen based on the experimental expectation that ISC occurs on a 100 fs time scale.^6^, ^7^ An energy‐based decoherence correction with a constant of C=0.1 a.u. was used^48^, ^49^ for the spin‐adiabatic states (states ordered by energy).^50^ To save computation time, gradients were only calculated for electronic states with an energy gap less than 0.3 eV. Otherwise, the defaults of the SHARC program package and the recently implemented SHARC/ADF interface^45^ have been used, where we calculate non‐adiabatic couplings approximately using wave‐function overlaps, as described in Ref. [51]. In the subsequent analysis, six trajectories had to be excluded from the 2NN ensemble due to computational problems in the simulations (see Supporting Information of Ref. [35]).

The propagation of the 75 trajectories for one of the NN derivatives during 500 fs required a total wall‐clock time of ca. 22500 h. Each trajectory was propagated on a single node with four Intel i5‐3470 cores and 16GB‐RAM, where ADF was run in parallel on 2 cores, i. e., allowing to calculate the gradients of two electronic states simultaneously.

### 2.4 Gas Phase Calculations and Solvent Effects

Experimental excited‐state dynamics studies of the NN derivatives were conducted in solvent.^2^, ^3^, ^4^, ^5^, ^6^, ^7^, ^8^ The experimental observations suggest that the main influence of the solvent results in small changes of the measured time constants by a factor up to 2. This suggests that, while there seems to be a solvent effect, it is secondary and does not alter the general reaction profile, but only affects the rates to a small extent. Thus, we believed it safe to neglect the solvent and carry out all calculations in gas phase.

### 2.5 Potential Energy Scan and Excited‐State Geometry Optimizations.

A relaxed potential energy scan along the nitro group torsional angle *γ* was performed for the ground states of all three NN derivatives. The scans were performed between $\left[0,90{}^{\circ }\right]$ in steps of $10{}^{\circ }$ . At each relaxed geometry, excited states were calculated using the same level‐of‐theory as in the dynamics. In addition, for each NN derivative, excited‐state geometry optimizations were performed for two excited states that later will be referred to as $S_{CT}\left(\pi \pi^{*}\right)$ and $S_{LE}\left(n\pi^{*}\right)$ . The geometry optimizations were carried out starting from structures selected from the trajectories, at which the corresponding state was the lowest‐energy excited singlet state (*S*_1_).

### 2.6 Transition‐Density Matrix Analysis

Electronically excited states are often described by referring to the (canonical) orbitals that characterize the most important configurations contributing to this electronic states. In cases where there is a large number of important configurations, this description can be incomplete and misleading. A better description can be achieved by analyzing the one‐particle transition‐density matrix. By singular‐value decomposition of the transition‐density matrix, one obtains a pair set of hole and electron natural transition orbitals (NTOs).^52^ This representation is more compact, as it often allows for a single pair of hole and electron NTOs to represent ∼99 % of the character of the excitation, which greatly simplifies the characterization. To describe excited states at a set of different geometries, it can though be inconvenient to use NTOs; as orbitals are oriented in space in different directions, it is not possible to compute meaningful ”averaged“ orbitals for the different geometries. In these cases, we found it convenient to characterize excited states in terms of the atomic contributions of the hole and electron parts of the transition density. Since these are atomic properties, their representation is invariant with respect to any nuclear displacements in the molecule, such as bond stretches or dihedral torsions, while comparison of both electron and hole parts yields a easily understandable illustration of the charge flow that occurs during the electronic transition. The analysis of transition‐density matrix has been performed using the TheoDORE program package.^52^, ^53^, ^54^

## 3 Results

### 3.1 Absorption Spectra and Initially Excited States

We begin our discussion by analyzing the absorption spectra of 2NN, 1NN, and 2M1NN. This analysis serves to determine the accuracy of the electronic structure level‐of‐theory and to establishes the character of the electronic states upon excitation. The calculated gas phase absorption spectra at the PBE0/DZP level of theory and their individual state contribution are shown in Figure 1 (a–c). Additionally, experimental spectra for 2NN and 1NN recorded in n‐heptane^55^ are shown, as well as for 2M1NN, for which the solvent is not reported.^56^ Other available spectra of 2M1NN in cyclohexane and acetonitrile^7^ only cover the range of 425–275 nm, but show similar features to the one in the unknown solvent.

We have previously calculated the excited states of 2NN in methanol at the PBE0/def2‐TZVP level‐of‐theory using different solvent and vibrational sampling methods.^35^ The corresponding calculated absorption spectra closely resemble the experimental absorption spectrum of 2NN in methanol and the positions of calculated and experimental absorption band maxima typically differ by less than 0.3 eV. In the present comparison between calculated absorption spectra in gas phase and experimental absorption spectra in n‐heptane or the unknown solvent, we find similar energy differences between the absorption bands. Thus, this suggests that the current level‐of‐theory is indeed able to describe the electronic structure of the excited states of the NN derivatives well. Notably, this energetic difference between the calculated and experimental absorption bands is smaller than 0.3 eV for the lowest‐energy absorption bands of the NN derivatives (2NN: 0.06 eV; 1NN: 0.1–0.2 eV; 2M1NN: 0.13 eV) where the excited‐state dynamics are initiated.

**Figure 2:**
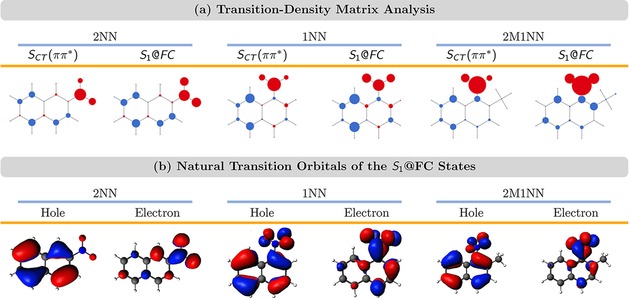
(a) Atomic hole‐electron difference population averaged for the electronic states initially excited in the dynamics simulations [*S_CT_* (*ππ✶*)] as well as for the *S*_1_ tate at the Franck‐Condon geometry (*S*_1_@*FC*). The size of the circles corresponds to the size of the hole‐electron difference population. The circles are blue (red) when the hole‐electron population is positive (negative), i. e., charge is transferred from blue to red circles when going from the ground to the excited state. (b) Natural‐transition orbitals characterizing the *S*_1_@*FC* states.

Similar to the dynamics investigated in the experimental studies,^2^, ^3^, ^4^, ^5^, ^6^, ^7^, ^8^ we start our nonadiabatic dynamics simulations by exciting to electronic states in the lowest‐energy absorption band corresponding to the energy ranges indicated in gray in Figure 1. The included electronic states are stochastically selected based on their oscillator strength to initiate the nonadiabatic dynamics simulations. In the molecular Coulomb Hamiltonian (MCH) representation, where electronic states are ordered according to their energy,^50^ these states correspond to the *S*_1_‐*S*_2_ (2NN), *S*_1_‐*S*_3_ (1NN), and *S*_1_‐*S*_4_ (2M1NN) states. The initially excited states possess similar spectroscopic character. This is demonstrated in Figure 2, where we show the average hole‐electron difference populations of the initially excited states as obtained from a transition‐density matrix analysis. The size of the circles corresponds to the size of the hole‐electron difference population. The circles are blue (red) when the hole‐electron difference population is positive (negative); in this representation negative charge is transferred from blue to red circles when going from the ground to the excited state. As can be seen in Figure 2, the initially excited states are described by $\pi \pi^{*}$ excitations that involve charge transfer from the aromatic ring system to the atoms of the nitro group [S${}_{CT}\left(\pi \pi^{*}\right)$ ]. Figure 2 also shows the atomic hole‐electron difference populations of the first excited state at the Franck‐Condon geometry ($S_{1}@FC$ ) for each NN derivative. As can be seen, these resemble closely to those of the S${}_{CT}\left(\pi \pi^{*}\right)$ states. Thus, we can conclude that the dynamics simulations will be initiated in an ensemble of $S_{CT}\left(\pi \pi^{*}\right)$ states which correspond to the $S_{1}@FC$ state.

### 3.2 Electronic State Populations

**Figure 3:**
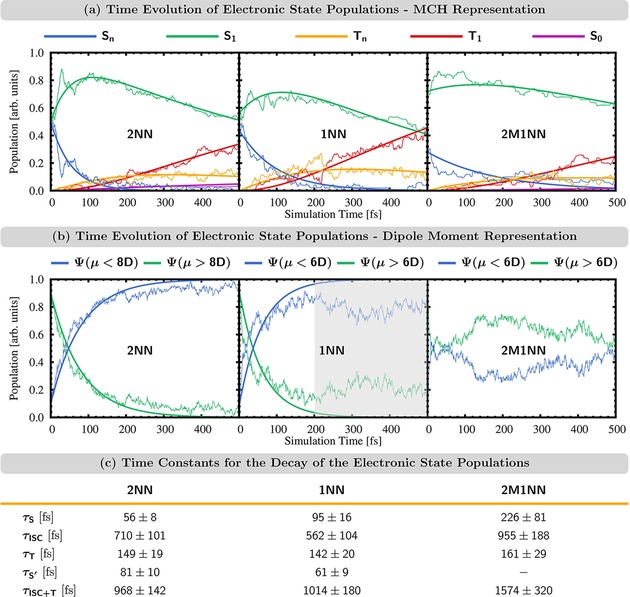
Time evolution of the electronic state populations. Thin lines represent actual state populations while thick lines represent fit functions. (a) MCH representation. Populations of higher‐excited singlet states *S_n_* (*n≥*2) and higher‐excited triplet states *T_n_* (*n≥*2) combined into one function each. (b) Dipole moment representation. Populations of all states with dipole moment above/below 8 D (2NN) or 6 D (1NN/2M1NN) combined into one function each. (c) Time constants for the exponential fit functions shown in (a/b) based on eqs. (1)–(3).

Figure 3 (a) shows the time evolution of the electronic state populations in the MCH representation for 500 fs. All three NN derivatives follow the same mechanism: first, the molecules undergo internal conversion (IC) from the higher‐lying singlet states *S_n_* to the *S*_1_, from which the molecules transfer to the higher‐lying triplet states *T_n_* via ISC, before they relax via IC in the triplet manifold to the *T*_1_ state. This mechanism can be described by the equation

(1) $$S_{n}\overset{\tau_{S}}{\underset{}{\to }}S_{1}\overset{\tau_{ISC}}{\underset{}{\to }}T_{n}\overset{\tau_{T}}{\underset{}{\to }}T_{1}.$$

Exponential fit functions based on this equation are shown in Figure 3 (a) (thick lines). Time constants for these fits are shown in Figure 3 (c) with errors estimated using the bootstrap method with a sample size of 100 copies.^57^ According to the fits based on eq. (1), the initial dynamics in the singlet manifold proceeds with time constants *τ_S_* of 56 fs, 95 fs, and 226 fs for 2NN, 1NN, and 2M1NN, respectively. Then, ISC from the singlet to the triplet manifold occurs with a time constants between 0.5–1.0 ps, while the final IC to the *T*_1_ in the triplet manifold occurs with time constants of ca. 150 fs, a similar time scale as the initial dynamics occurring in the singlet manifold.

The fit functions in Figure 3 (a) closely follow the time evolution of the actual electronic state population (thin lines). This demonstrates that the simple mechanism in eq. (1) is sufficient to explain the excited‐state dynamics. However, in the MCH representation, we cannot distinguish between states with the same spin but different electronic character. To obtain more insight within a spin manifold, we have to monitor the time evolution of different properties. In 2NN, we had found^35^ that the majority of singlet‐to‐triplet transitions do not occur from the initially excited $S_{CT}\left(\pi \pi^{*}\right)$ state but from a locally excited $n\pi^{*}$ singlet state [$S_{LE}\left(n\pi^{*}\right)$ ]. Then, the initial dynamics in the singlet manifold could be described by

(2) $$S_{CT}\left(\pi \pi^{*}\right)\overset{\tau_{S{}^{'}}}{\underset{}{\to }}S_{LE}\left(n\pi^{*}\right).$$

The $S_{CT}\left(\pi \pi^{*}\right)$ and $S_{LE}\left(n\pi^{*}\right)$ states of 2NN possess quite different dipole moments μ. For example, at their respective minimum‐energy geometries, $\mu [S_{CT}\left(\pi \pi^{*}\right)]=12.9$ D and $\mu [S_{LE}\left(n\pi^{*}\right)]=3.7$ D (see Figure 5). Thus, to analyze the initial dynamics in the singlet manifold, we chose an arbitrary intermediate threshold value for the dipole moment (μ=8 D) and counted every state with μ>8 D/μ<8 D as a state of $S_{CT}\left(\pi \pi^{*}\right)$ /$S_{LE}\left(n\pi^{*}\right)$ character. Following this classification, the time evolution of the electronic state populations and a fit function based on eq. (2) are shown in Figure 3 (b) for 2NN. The fit function possesses a time constant of $\tau_{S{}^{'}}=81$ fs [Figure 3 (c)] and closely follows the electronic state populations, thus, demonstrating that the initial dynamics in the singlet manifold of 2NN are well described by eq. (2).

**Figure 4:**
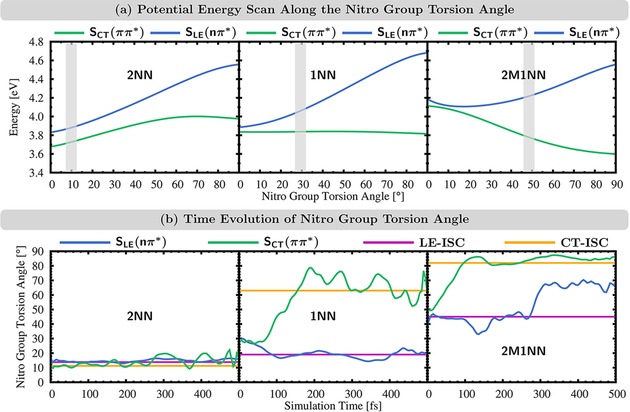
(a) Excited‐state energies along a relaxed potential energy scan of the nitro group torsion angle *γ*. Gray areas highlight average *γ* (plus standard deviation) in the initial conditions. (b) Time evolution of the averaged nitro group torsion angle *γ* for trajectories in either *S_LE_* (*nπ*✶) or *S_CT_* (*ππ*✶) state and average *γ* for all ISC transition geometries of the LE and CT pathways.

**Figure 5:**
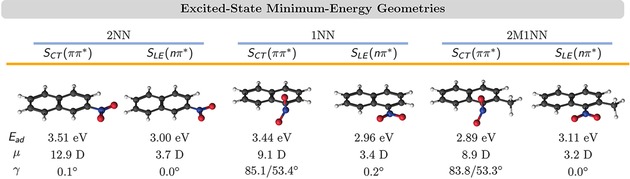
Excited‐state minimum‐energy geometries of the *S_CT_* (*ππ*✶) and *S_LE_* (*nπ*✶) states. Adiabatic excitation energies *E_ad_* with respect to the ground‐state minimum‐energy geometries. *μ* and *γ* denote excited‐state dipole moments and the nitro group torsion angle, respectively. Both states are the lowest‐energy excited singlet state at their respective minimum‐energy geometry.

For 1NN and 2M1NN, we attempted the same analysis. As will be discussed in section 3.3, also for 1NN and 2M1NN large parts of the ISC processes occur from a $S_{LE}\left(n\pi^{*}\right)$ state, suggesting that the initial dynamics of 1NN and 2M1NN also follow eq. (2). For both molecules, the $S_{LE}\left(n\pi^{*}\right)$ states have dipole moments of 3.2–3.4 D at their minimum‐energy geometries (see Figure 5), similar to the μ=3.7 D of the $S_{LE}\left(n\pi^{*}\right)$ state of 2NN.

The $S_{CT}\left(\pi \pi^{*}\right)$ states of 1NN and 2M1NN have dipole moments of ca. 9 D, somewhat smaller than that of the $S_{CT}\left(\pi \pi^{*}\right)$ state of 2NN (12.9 D). The observation that the dipole moment of the $S_{CT}\left(\pi \pi^{*}\right)$ state is smaller for 1NN and 2M1NN compared to 2NN can be explained by considering the geometries of the three molecules. In 1NN and 2M1NN, the nitro group is closer to the center of the aromatic ring system than in the case of 2NN. This results in a smaller dipole moment when the same amount of charge is transferred from the ring the nitro group – as is the case for the $S_{\mathrm{CT}}\left(\pi \pi^{*}\right)$ state.

The smaller difference between the dipole moments of $S_{\mathrm{CT}}\left(\pi \pi^{*}\right)$ ‐type and $S_{\mathrm{LE}}\left(n\pi^{*}\right)$ ‐type states of 1NN and 2M1NN complicates the discrimination between these states along the dynamics. Using a threshold value of μ=6 D, the evolution of the electronic state populations for 1NN and 2M1NN is shown in Figure 3 (b). For 1NN, the population of the Ψ(μ>6D) state (thin green line) decays exponentially until ca. 200 fs, after which the population starts to oscillate. This indicates that at these later times, different reactions add to the change of electronic state populations in this dipole moment representation. Based on this observation, we calculated a fit function for eq. (2) using only the data until t=200 fs, which yields a time constant of $\tau_{S{}^{'}}=61\pm 9$ fs for IC within the singlet manifold of 1NN. For 2M1NN, no meaningful fit function could be obtained that agreed reasonably with the time evolution of the excited‐state populations of the dynamics – neither using the threshold of μ=6 D [shown in Figure 3 (b)] nor any other similar value. As discussed at the end of the next section, this could be due to an equilibrium between both singlet states [$S_{\mathrm{CT}}\left(\pi \pi^{*}\right)\overset{⇀}{↽}S_{\mathrm{LE}}\left(n\pi^{*}\right)$ ], in which case it would not be possible to calculate a time constant.

We conclude by pointing out that the time constant of the triplet IC (*τ_T_*) is much smaller than that of the preceding ISC (*τ_ISC_*) in all three NN derivatives, meaning that the slow ISC reaction is followed by a fast IC process. While both processes can be well characterized in the theoretical simulations, it can be difficult to monitor them separately in experiment. The resolution of the fast second process may be lost, which can lead to both reactions being interpreted as a one, and the measurement of only a single, effective time constant. Thus, to later on compare our computational results to the results obtained from by time‐resolved experiments, we also define an effective time constant *τ*_*ISC*+*T*_ that combines the slow singlet‐to‐triplet ISC time constant *τ_ISC_* with the fast, subsequent triplet IC time constant *τ_T_*. This is done via

(3) $$S_{n}\overset{\tau_{S}}{\underset{}{\to }}\mathrm{Intermediate}\;\mathrm{Species}\left(S_{1},T_{n}\right)\overset{\tau_{ISC+T}}{\underset{}{\to }}T_{1},$$

The effective time constants *τ*_*ISC*+*T*_ are listed in Figure 3 (c).

### 3.3 Electronic States Involved in Intersystem Crossing

**Figure 6:**
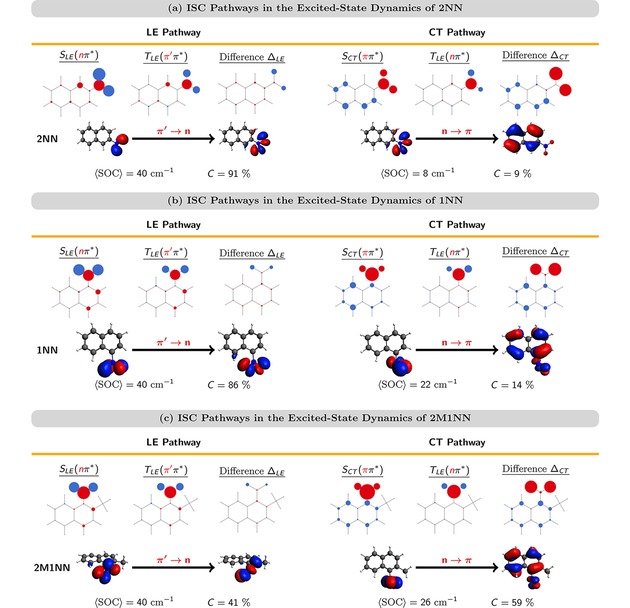
Characterization of ISC pathways of the nitronaphthalene derivatives 2NN (a), 1NN (b), and 2M1NN (c). Atomic hole‐electron difference populations for the singlet and triplet states involved in the ISC pathways as well as the difference between the populations of both states ▵_*LE/CT*_ . Below: NTOs involved in the ISC transition. $\left\langle \mathrm{SOC}\right\rangle$ and *C* denote the size of the average spin‐orbit coupling and the contribution of the ISC pathway, respectively.

In 2NN^35^, ^36^, we found two ISC pathways operative at T=300 K: one involving a transition from the $S_{LE}\left(n\pi^{*}\right)$ to a $T_{LE}\left(\pi {}^{'}\pi^{*}\right)$ state, and one from the initially excited $S_{CT}\left(\pi \pi^{*}\right)$ to a $T_{LE}\left(n\pi^{*}\right)$ state. These pathways were labeled ”major“ and ”minor“ according to their relative contributions to the total ISC yield (91 vs 9 %),^35^ and labeled as LE and CT pathway, respectively, in Ref. [36]. The atomic hole‐electron difference populations of the four excited states of 2NN that describe the LE and CT ISC pathways are shown in Figure 6 (a). Note that the labeling of the LE and CT pathways does not refer to the singlet states $S_{LE}\left(n\pi^{*}\right)$ and $S_{CT}\left(\pi \pi^{*}\right)$ from which the system transfers to the triplet manifold, but to the characters of the ISC transitions themselves. The $S_{LE}\left(n\pi^{*}\right)\to T_{LE}\left(\pi {}^{'}\pi^{*}\right)$ transition is a transition localized at the nitro group, while the $S_{CT}\left(\pi \pi^{*}\right)\to T_{LE}\left(n\pi^{*}\right)$ is a transition involving substantial charge transfer.

The four excited states $S_{LE}\left(n\pi^{*}\right)$ , $T_{LE}\left(\pi {}^{'}\pi^{*}\right)$ , $S_{CT}\left(\pi \pi^{*}\right)$ , and $T_{LE}\left(n\pi^{*}\right)$ are described by the same electron NTO ($\pi^{*}$ ), but differ in their hole NTOs (*n*, $\pi {}^{'}$ , *π*). For these reasons, the $S_{LE}\left(n\pi^{*}\right)\to T_{LE}\left(\pi {}^{'}\pi^{*}\right)$ transition of the LE pathway can be described by transferring the hole from the *n* to the $\pi {}^{'}$ orbital or, equivalently, to a $\pi {}^{'}\to n$ transition of an electron. In the same manner, the $S_{CT}\left(\pi \pi^{*}\right)\to T_{LE}\left(n\pi^{*}\right)$ transition of the CT pathway is described by a either a π→n transition of a hole or a n→π transition of an electron. To illustrate this, in Figure 6(a) the ISC transitions in terms of the hole NTOs are shown for a sample geometry. In addition, Figure 6(a) reports the average size of the spin‐orbit coupling $\left\langle \mathrm{SOC}\right\rangle$ between the corresponding singlet and triplet states (40 and 8 cm^−1^) as well as the relative contributions of both pathways to the total ISC yield (91 vs 9 %) for the LE and CT pathway, respectively.

In 1NN and 2M1NN, ISC proceeds similarly to 2NN via an $S_{LE}\left(n\pi^{*}\right)\to T_{LE}\left(\pi {}^{'}\pi^{*}\right)$ transition (LE pathway) and an $S_{CT}\left(\pi \pi^{*}\right)\to T_{LE}\left(n\pi^{*}\right)$ transition (CT pathway). The main characteristics of both pathways of 1NN and 2M1NN are shown in Figure 6 (b,c). As can be seen, both the atomic hole‐electron difference populations of the singlet and triplet states involved in the ISC transitions as well as the hole NTOs of 1NN and 2M1NN resemble closely those of the ISC pathways of 2NN. For 1NN, the majority of ISC occurs via the LE pathway (86 %), while a smaller fraction of ISC (14 %) occurs via the CT pathway, which is similar to the ISC contributions of 2NN (91 vs. 9 %). In contrast, a majority of 59 % of ISC events occurs via the CT pathway while the LE pathway contributes only 41 % to the total ISC yield.

To rationalize the difference between the relative contributions of the LE and CT pathways for 2M1NN compared to 2NN and 1NN, we investigated why the CT pathway might become more favorable in 2M1NN. We found two factors that can explain this observation: an increase of the spin‐orbit coupling between the $S_{CT}\left(\pi \pi^{*}\right)/T_{LE}\left(n\pi^{*}\right)$ states and the behavior of the nitro group torsion during the dynamics. This first factor can be seen in Figure 6, which reports the average size of spin‐orbit couplings $\left\langle SOC\right\rangle$ between the $S_{LE}\left(n\pi^{*}\right)/T_{LE}\left(\pi {}^{'}\pi^{*}\right)$ and $S_{CT}\left(\pi \pi^{*}\right)/T_{LE}\left(n\pi^{*}\right)$ pairs, calculated at geometries where the ISC transitions occur. As can be seen, the average spin‐orbit coupling for the LE pathway is $\left\langle \mathrm{SOC}(\mathrm{LE})\right\rangle =40$ cm^−1^ for all three NN derivatives. In contrast, $\left\langle \mathrm{SOC}(\mathrm{CT})\right\rangle$ for the CT pathway increases from ca. 8 cm^−1^ for 2NN to 22 and 26 cm^−1^ for 1NN, and 2M1NN, respectively. This means that the probability for ISC via a $S_{CT}\left(\pi \pi^{*}\right)\to T_{LE}\left(n\pi \pi^{*}\right)$ transition is increased for both 1NN and 2M1NN once these molecules reach suitable geometries.

In addition to the increase of $\left\langle \mathrm{SOC}(\mathrm{CT})\right\rangle$ , we find that the $S_{CT}\left(\pi \pi^{*}\right)\to T_{LE}\left(n\pi^{*}\right)$ transitions occur at geometries with larger nitro group torsional angles *γ* : $\gamma_{CT}=11\pm 2{}^{\circ }$ (2NN), $\gamma_{CT}=63\pm 5{}^{\circ }$ (1NN), and $\gamma_{CT}=83\pm 2{}^{\circ }$ (2M1NN). Inspecting the *n* and *π* hole NTOs describing the CT pathway, we find that at larger nitro group torsion angles *γ* there is an admixture of *n*‐type orbitals to the *π* NTO. This can be seen for the exemplary hole NTOs for the CT pathway for 1NN and 2M1NN in Figure 6 (b,c). At small values of *γ*, there is no admixture visible in the orbitals and at planar geometries the *n* and *π* hole NTO are completely separated at the nitro group and the aromatic ring, respectively – see Figure 6 (a) for the case of 2NN. Thus, it seems that the admixture of *n* and *π* orbitals at geometries with larger torsion *γ* allows for larger SOC.

For the LE pathway, we find that the $S_{LE}\left(n\pi^{*}\right)/T_{LE}\left(\pi {}^{'}\pi^{*}\right)$ crossing occurs at geometries with nitro group torsion of $\gamma_{LE}=13\pm 1{}^{\circ }$ (2NN), $\gamma_{LE}=20\pm 1{}^{\circ }$ (1NN), and $\gamma_{LE}=45\pm 3{}^{\circ }$ (2M1NN). Thus, the $S_{LE}\left(n\pi^{*}\right)\to T_{LE}\left(\pi {}^{'}\pi^{*}\right)$ transitions occur at geometries with larger *γ* in the order 2NN<1NN<2M1NN . Despite the different *γ_LE_*, $\left\langle \mathrm{SOC}(\mathrm{LE})\right\rangle$ is the same for all three NN derivatives – in contrast to the CT pathway. This is because, the LE pathway is described by a $\pi {}^{'}\to n$ transition where both orbitals are located solely on the nitro group. Thus, their relative orientation is independent of the orientation of the nitro group, as can be seen for the exemplary $\pi {}^{'}$ and *n* orbitals of the LE pathway shown in Figure 6(a)–(c).

### 3.4 Role of the Nitro Group Torsion

The larger $\left\langle \mathrm{SOC}(\mathrm{CT})\right\rangle$ at the ISC geometries of 1NN and 2M1NN may explain an increase of the CT contribution for both molecules. However, it does not explain why the CT pathway becomes the major reaction channel of ISC for 2M1NN, since $\left\langle \mathrm{SOC}(\mathrm{CT})\right\rangle =26$ cm^−1^ is still smaller than $\left\langle \mathrm{SOC}(\mathrm{LE})\right\rangle =40$ cm^−1^. To rationalize this, we consider the potential energies curves of the $S_{LE}\left(n\pi^{*}\right)$ and $S_{CT}\left(\pi \pi^{t}\right)$ states as a function of the nitro group torsion *γ*, see Figure 4 (a). The average *γ* in the initial geometries is shown in gray areas. The dynamics of 2M1NN start at geometries around $\gamma =48{}^{\circ }$ , where the initially excited $S_{CT}\left(\pi \pi^{*}\right)$ state is ca. 0.4 eV below the $S_{LE}\left(n\pi^{*}\right)$ state. The gradient of the $S_{CT}\left(\pi \pi^{*}\right)$ energy is directed towards larger values of *γ*, and the energy becomes minimal at $90{}^{\circ }$ where the energetic separation between both states is maximal. This suggests that initially, the trajectories remain in the $S_{CT}\left(\pi \pi^{*}\right)$ state and move towards geometries with larger *γ* closer to the geometries where ISC occurs through the CT pathway ($\gamma ∼83{}^{\circ }$ ). The fact that the minimum of the $S_{CT}\left(\pi \pi^{*}\right)$ state (Figure 5) lies 0.2 eV below the planar $S_{LE}\left(n\pi^{*}\right)$ minimum and is characterized by a large nitro group torsional angle supports this suggestion.

Nevertheless, still 41 % of the ISC of 2M1NN occurs via the LE pathway. Thus, the 2M1NN trajectories seem to contain enough energy to transfer at least partially to the $S_{LE}\left(n\pi^{*}\right)$ state. This suggests an equilibrium between the $S_{CT}\left(\pi \pi^{*}\right)$ and $S_{LE}\left(n\pi^{*}\right)$ states that is then drained by ISC. This equilibrium would explain why the simple $S_{CT}\left(\pi \pi^{*}\right)\to S_{LE}\left(n\pi^{*}\right)$ model [eq. (2)] used for 2NN and 1NN did not allow for a reasonable description of the initial dynamics in the singlet manifold for 2M1NN [Figure 3 (b)]. In that analysis, the trajectories were characterized as either $S_{CT}\left(\pi \pi^{*}\right)$ or $S_{LE}\left(n\pi^{*}\right)$ when their dipole moment μ is above or below 6 D which lies intermediate between the dipole moments of the $S_{CT}\left(\pi \pi^{*}\right)$ or $S_{LE}\left(n\pi^{*}\right)$ states at their respective minimum‐energy geometries. Using the same dipole moment criterion for characterization, we analyzed the time evolution of the nitro group torsion angle *γ*, see Figure 4 (b). As can be seen, for 2M1NN, trajectories in the $S_{CT}\left(\pi \pi^{*}\right)$ state (μ>6 D) move from initial angles around $50{}^{\circ }$ towards larger angles around 80–90°, which is close to the average torsional angle of the CT pathway ($\gamma =82{}^{\circ }$ ). In contrast, trajectories in the $S_{LE}\left(n\pi^{*}\right)$ state (μ<6 D) stay for the first 300 fs close to the average angle of the LE pathway $\left(\gamma =45{}^{\circ }\right)$ .

For the sake of completeness, we show the potential energy scan for 2NN and 1NN in Figure 4 (a). We note that the average nitro group torsion angle of 2NN in the initial conditions ($\gamma =10{}^{\circ }$ ) is not that of the minimum‐energy geometry (γ=0 ). Due to the symmetry of the molecule, torsion of the nitro group away from a *C*_s_‐symmetric minimum‐energy ground state to either direction leads to pairs of enantiomers with equivalent physical properties.^58^, ^59^ Thus, rather than using any single CCNO dihedral angles, we take the average of the four CCNO dihedral angles to represent the nitro group torsion *γ*, which results in an average $\gamma =10{}^{\circ }$ for the initial conditions.

Finally, we also show the time evolution of the nitro group torsion angle for the dynamics of 2NN and 1NN in Figure 4 (b). For 2NN, all trajectories in either $S_{CT}\left(\pi \pi^{*}\right)$ or $S_{LE}\left(n\pi^{*}\right)$ state stay at nitro group torsion angles between 10–20°, i. e., close to the average torsion angles of the CT and LE pathways of 11 and 14°, respectively. In contrast, for 1NN, the trajectories in $S_{CT}\left(\pi \pi^{*}\right)$ or $S_{LE}\left(n\pi^{*}\right)$ split up towards larger and smaller torsion angles, respectively. This brings both sets of 1NN trajectories closer to geometries where they can undergo ISC. Starting around $\gamma =30{}^{\circ }$ , trajectories in the $S_{CT}\left(\pi \pi^{*}\right)$ state drive towards the average angle of the CT pathway ($\gamma =63{}^{\circ }$ ) whereas trajectories in the $S_{LE}\left(n\pi^{*}\right)$ state drive towards the average angle of the LE pathway ($\gamma =20{}^{\circ }$ ).

## 4 Previous Studies

**Figure 7:**
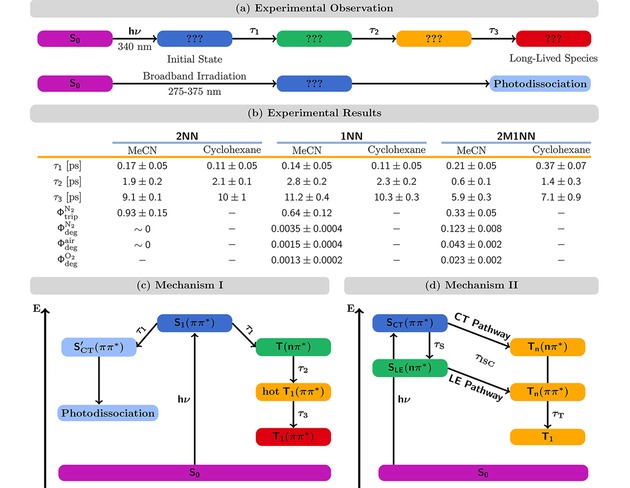
(a) Summary of experimental observations. (b) Summary of experimental results of excited‐state dynamics studies.^[5–8]^ Time constants *τ* taken from transient absorption experiments. Φ_trip_ and Φ_deg_ denote the triplet and photodegradation quantum yields, respectively, for experiments conducted in acetonitrile saturated with either N_2_, air, and O_2_ (see superscripts). (c) Mechanism based on transient absorption experiment and static quantum chemical calculations by the groups of Peon and Crespo‐Hernández.^2^, ^3^, ^4^, ^5^, ^6^, ^7^, ^8^ (d) Mechanism based on our previous nonadiabatic dynamics simulations^[35,36]^ as well as this study.

This section reviews previous experimental and theoretical results on the NN derivatives. Figure 7 collects kinetic models and time constants available from the literature. Using femtosecond fluorescence up‐conversion and transient absorption spectroscopy experiments the groups of Peon and Crespo‐Hernández^2^, ^3^, ^4^, ^5^, ^6^, ^7^, ^8^ observed a sequence of three decays of signals from different electronic states (depicted in blue, green, orange), before a long‐lived state (red) is populated (Figure 7a).

The molecules were excited with light around the maximum of the lowest‐energy absorption band or its lower‐energy arm. The experiments were conducted in N_2_‐saturated solutions of various polar and non‐polar solvents. For all experimental signals, time constants of similar size were determined for the three sequential decays. For our discussion, we show exemplary time constants from transient absorption experiments in acetonitrile (MeCN) and cyclohexane^6^ in Figure 7 (b) after excitation at 340 nm, as for this setting, the most data is available.

The first assignment was made by Peon and co‐workers based on fluorescence up‐conversion experiments and it has been the most crucial: *τ*_1_ was assigned to singlet‐to‐triplet ISC for 1NN.^2^ This assignment was inspired by early semi‐empirical calculations^59^ that predicted a triplet state to be close in energy with the initially excited singlet state. This triplet state was of 50 % $\sigma \pi^{*}$ character, which following El‐Sayed's rules is a possible candidate for ISC from a singlet $\pi \pi^{*}$ state. It was known^2^ that NPAHs can possess quite high triplet quantum yields, e. g., $\Phi_{\mathrm{trip}}=0.63\pm 0.10$ for 1NN. Thus, the molecules had to undergo ISC as excited‐state populations end up in a triplet state. It was found later on that the lifetime of the long‐lived (red) species of 1NN decreases considerably when performing the experiments under aerobic conditions.^5^

In their fluorescence up‐conversion experiments, Peon and co‐workers also investigated other NPAHs besides 1NN.^2^ For 1NN, excitation at 385 nm gave a signal with a mono‐exponential decay. For the other NPAHs, however, this excitation lead to a signal with double‐exponential decay with a fast component on the sub‐100 fs time scale and a slower component on the ps time scale. The values for the other NPAH derivatives were of similar magnitudes as *τ*_1_ and *τ*_2_ for the NN derivatives later found in transient‐absorption experiments. In contrast to 1NN, for the other NPAHs investigated, the sub‐100 fs decay was not assigned to ISC but instead was suggested to correspond to conformational dynamics in the singlet state.

Based on TDDFT calculations, the nature of the ISC channel was assigned for 1NN.^3^ A triplet state of $n\pi^{*}$ character [$T_{n}\left(n\pi^{*}\right)$ ] was found close in energy to the lowest‐energy $\pi \pi^{*}$ singlet state at the FC geometry [$S_{1}\left(\pi \pi^{*}\right)$ ], while the lowest‐energy triplet state at the FC geometry was of $\pi \pi^{*}$ character [$T_{1}\left(\pi \pi^{*}\right)$ ]. Thus, it seemed natural to suggest that ISC occurs via $S_{1}\left(\pi \pi^{*}\right)\to T_{n}\left(n\pi^{*}\right)$ followed by relaxation in the triplet manifold via $T_{n}\left(n\pi^{*}\right)\to T_{1}\left(\pi \pi^{*}\right)$ .

Following the assignment of *τ*_1_ and the long‐lived species, the other two decay reactions with time constants *τ*_2_ and *τ*_3_ were assigned to relaxation within in triplet manifold.^4^, ^5^ Interestingly, Peon and co‐workers remarked that the picosecond relaxation time to the *T*_1_ appeared to be significantly slower than what is usually observed for IC processes in singlet excited states.^4^ Crespo‐Hernández and co‐workers assigned only the faster time constant $\tau_{2}∼1$ ps to IC in the triplet manifold, leading to a hot *T*_1_ state, while the larger $\tau_{3}∼10$ ps described the subsequent vibrational cooling from the hot *T*_1_.^5^

Crespo‐Hernández and co‐workers then addressed the triplet quantum yield of 1NN, that is (only) 0.64 in MeCN, despite the very large ISC rate. Following the assumption that $S_{1}\to S_{0}$ IC was negligible – a statement made by Peon and co‐workers earlier, albeit, for two other NPAHs,^2^ not for 1NN – Crespo‐Hernández and co‐workers proposed the existence of another nonradiative decay channel competing with ISC on a 100 fs time scale.^5^ This channel was supposed to lead to dissociation of the nitro group through torsion in the $S_{1}\left(\pi \pi^{*}\right)$ state to a charge‐transfer state [${S{}^{'}}_{CT}\left(\pi \pi^{*}\right)$ ]. Thus, the excited‐state dynamics should bifurcate after excitation to the bright $S_{1}\left(\pi \pi^{*}\right)$ state: part of the electronic population would reach the triplet manifold via a $T_{n}\left(n\pi^{*}\right)$ state, while another part accumulates in ${S{}^{'}}_{CT}\left(\pi \pi^{*}\right)$ from which the molecule dissociates. As both processes would happen on a similar time scale, only one time constant would be observed in experiment, *τ*_1_. These assumptions are summarized as Mechanism I in Figure 7 (c).

In later studies, Crespo‐Hernández and co‐workers investigated the excited‐state dynamics of 2NN and 2M1NN using transient absorption spectroscopy. They determined triplet quantum yields $\Phi_{\mathrm{trip}}$ and photodegradation quantum yields $\Phi_{\deg}$ for all three NN derivatives.^6^, ^7^, ^8^ The time‐resolved experiments yielded similar time constants for 2NN and 2M1NN as for 1NN [see Figure 7 (b)], which suggests that all three NN derivatives possess the same mechanism [Figure 7 (c)]. More significant differences were found for the quantum yields $\Phi_{\mathrm{trip}}$ and $\Phi_{\deg}$ . For 2NN, $\Phi_{\mathrm{trip}}=93\pm 15\%$ while the $\Phi_{\deg}∼0$ , i. e., 2NN was shown to be photostable suggesting that only ISC occurs after excitation. For 1NN, where $\Phi_{\mathrm{trip}}=64\pm 12\%$ , photodegradation was found to occur with $\Phi_{\deg}=0.35\%$ , while for2M1NN, $\Phi_{\mathrm{trip}}=33\pm 5\%$ and $\Phi_{\deg}=12.3\%$ . It was observed that, taking into account all three NN derivatives, there is an inverse relationship between $\Phi_{\mathrm{trip}}$ and $\Phi_{\deg}$ : $\Phi_{\deg}$ increased while $\Phi_{\mathrm{trip}}$ decreased when going from 2NN to 1NN to 2M1NN. This was taken as confirmation^6^, ^7^ of the competition between the ISC channel and the dissociative reaction channel proposed earlier.

Two CASSCF/CASPT2 studies by Canuto, Peon, and co‐workers^28^ as well as Giussani^30^ computed the minimum‐energy paths of some of the excited states of 1NN. These studies suggested that after excitation to the $S_{1}@FC$ state, the molecule moves towards the minimum of that state, where a triplet state with SOCs of ca. 65 cm^−1^ lies close in energy – either 0.11 eV below^28^ or 0.17 eV above.^30^ In both studies, the singlet and triplet state resemble the $S_{LE}\left(n\pi^{*}\right)$ and $T_{LE}\left(\pi \pi^{*}\right)$ states identified in this work. However, a direct comparison of the character of the states is difficult, as the wave functions of the electronic states were not fully reported. For example, the $n\pi^{*}$ configuration of the singlet state possesses only a weight of 45 % in the total wave function in the CASSCF/CASPT2 calculation of Ref. [30], while the NTO pair of the *n* and *π* orbitals in the $S_{LE}\left(n\pi^{*}\right)$ state accounts for 99 % of the state vector. Ref. [28] reports no details on the contribution of the electronic state wave functions.

## 5 Discussion

In the time‐resolved experiments,^2^, ^3^, ^4^, ^5^, ^6^, ^7^, ^8^ the NN derivatives are excited to the lowest‐energy absorption band, supposedly corresponding to the $S_{1}\left(\pi \pi^{*}\right)$ state at the FC geometry. Our initial conditions calculations confirm that the initial excitation populates only $S_{CT}\left(\pi \pi^{*}\right)$ states. However, at some geometries this state corresponds to the *S*_2_ or even the *S*_3_. Note that for excitation to the higher‐energy absorption bands of 2NN,^40^ a mixing of states corresponding to bright and dark states at the FC geometry is encountered.

Based on the presence of a close‐lying $T_{n}\left(n\pi^{*}\right)$ state at the FC geometry and the fact that the NN derivatives end up in long‐lived (triplet) states, it has been suggested that the molecules immediately undergo ISC.^2^, ^3^, ^4^, ^5^, ^6^, ^7^, ^8^ This lead to the assignment of first decay signal with time constant *τ*_1_ to ISC. Our simulations show, however, that the NN derivatives initially stay in the singlet manifold: 2NN and 1NN mainly relax from the initially excited $S_{CT}\left(\pi \pi^{*}\right)$ state to the $S_{LE}\left(n\pi^{*}\right)$ state with time constants $\tau_{S{}^{'}}=81\pm 10$ and 61±9 fs, respectively. These values for $\tau_{S{}^{'}}$ agree with the experimental values for *τ*_1_ for 2NN and 1NN in cyclohexane (110±50 fs), whereas the experimental values of *τ*_1_ in acetonitrile are slightly larger, 170±50 fs for 2NN and 140±50 fs for 1NN. For 2M1NN, our analysis suggests that part of the trajectories also undergo IC from the $S_{CT}\left(\pi \pi^{*}\right)$ state to the $S_{LE}\left(n\pi^{*}\right)$ state with geometries displaying nitro torsional angles *γ* similar to that of the FC geometry. The other 2M1NN trajectories that stay in the $S_{CT}\left(\pi \pi^{*}\right)$ state move towards geometries where the nitro group is nearly perpendicular to the aromatic ring ($\gamma ∼83{}^{\circ }$ ).

Attempts to calculate a time constant for the process of trajectories going from the $S_{CT}\left(\pi \pi^{*}\right)$ to the $S_{LE}\left(n\pi^{*}\right)$ state for 2M1NN failed, suggesting that there is an equilibrium between both states. A simple analysis of the time evolution of the adiabatic electronic states of 2M1NN provides a time constant of $\tau_{S}=226\pm 81$ fs for the relaxation process $S_{n}\to S_{1}$ . This *τ_S_* for 2M1NN is larger than those found for 2NN (56±8 fs) and 1NN (95±16 ). Conspicuously, the experimental decay time constant *τ*_1_ is also larger for 2M1NN (370±70 fs in cyclohexane and 210±50 fs in acetonitrile) than for 2NN and 1NN. It is thus tempting to identify the experimental increase of *τ*_1_ in 2M1NN with the calculated increase of *τ_S_*. Overall, we believe it is justified to assign the experimental time constant *τ*_1_ to nonadiabatic dynamics within the singlet manifold rather than to ISC to the triplet states.

Having made our case for the re‐assignment of the experimental time constant *τ*_1_, we now turn to *τ*_2_, that was previously assigned to IC within in the triplet manifold.^2^, ^3^, ^4^, ^5^, ^6^, ^7^, ^8^ Our simulations show that the second process instead is ISC which occurs with time constants *τ_ISC_* of ca. 550–950 fs. This then is followed by IC from the higher‐lying *T_n_* states to the *T*_1_ that occurs with a time constant of $\tau_{T}∼150$ fs for all NN derivatives. As discussed earlier, the resolution of a faster second process (*τ_T_*) after an initial slow process (*τ_ISC_*) might be lost in experiment. Thus, we defined an effective time constant *τ*_*ISC*+*T*_ including both ISC and IC within the triplet manifold to compare to *τ*_2_ instead. The effective time constants for 2NN and 1NN from the simulations are $\tau_{ISC+T}1.0\pm 0.1$ and 1.0±0.2 ps, respectively. The experimental time constants are $\tau_{2}∼2.0$ ps for 2NN and $\tau_{2}=2.3$ –2.8 ps for 1NN in cyclohexane and acetonitrile, respectively.^7^ Thus, the processes in the simulations are faster by a factor of 2–3. This might partially be due to the lack of solvent, which presents dissipation of the excess energy of the molecule to the environment, which would gradually slow down the dynamics. Nevertheless, the time constants *τ*_*ISC*+*T*_ and *τ*_2_ are of the same order of magnitude, so that it seems reasonable to suggest that both describe the same process.

For 2M1NN, our simulations predict $\tau_{ISC+T}=1.6\pm 0.3$ ps, that is, ISC is slower in 2M1NN than in 2NN and 1NN. In contrast, the experimental time constants for 2M1NN are $\tau_{2}=1.4\pm 0.3$ and 0.6±0.1 ps in cyclohexane and MeCN, respectively. The value of $\tau_{2}=1.4\pm 0.3$ ps from the experiments in cyclohexane is suspiciously close to our value of $\tau_{ISC+T}=1.6\pm 0.3$ ps calculated in gas phase. However, following our reasoning above for 2NN and 1NN, this should be seen as a fortuitous coincidence; rather, we should stick to our argument that both *τ*_*ISC*+*T*_ and *τ*_2_ are of the same of order of magnitude. More interestingly, however, for 2M1NN in MeCN, it was found that $\tau_{2}=0.6\pm 0.1$ ps. Thus, if *τ*_2_ can be assigned to ISC, this would be experimental evidence for sub‐picosecond ISC in a NN derivative. This assignment of *τ*_2_ for ISC must not necessarily be done for 2M1NN, even though we argued for the same assignment in the cases of 2NN and 1NN. For 2NN and 1NN, experiments in MeCN showed that the majority of the excited‐state populations ended up in triplet states with triplet quantum yields of $\Phi_{\mathrm{trip}}^{N_{2}}=0.93\pm 0.15$ and 0.64±0.12 , respectively. In contrast, in 2M1NN, the triplet quantum yield amounted to $\Phi_{\mathrm{trip}}^{N_{2}}=0.33\pm 0.05$ , that is, only a minority of the excited‐state population ended up in a triplet state. Thus, from these results, it is not clear, whether *τ*_2_ describes the same process in all three NN derivatives. In line with the smaller triplet quantum yield of 2M1NN, we find that ISC is also slower in 2M1NN than in 2NN and 1NN.

The third experimental time constant *τ*_3_ was assigned to vibrational cooling in the *T*_1_ state.^7^ The experimental values of $\tau_{3}=6-11$ ps are too large to be reproduced by our 500 fs simulations, and, thus, we decline to draw any conclusions.

Previous studies^2^, ^3^, ^4^, ^5^, ^6^, ^7^, ^8^, ^28^, ^30^ considered only one ISC pathway going from the *S*_1_ state directly to a triplet state of the correct symmetry according to El‐Sayed's rules. In contrast, our simulations showed two distinct ISC pathways: the LE and the CT pathway. The CT pathway corresponds to the pathway initially considered by the groups of Peon and Crespo‐Hernández based on their static TDDFT calculations,^2^, ^3^, ^4^, ^5^, ^6^, ^7^, ^8^ while the LE pathway appears to be the one found in the CASSCF/CASPT2 studies.^28^, ^30^

The last process occurring in our dynamics simulation is IC within the triplet manifold. This process occurs on a time scale of the order of hundreds of femtoseconds, analogously as the IC that occurs initially within the singlet manifold.

Following the assumption^6^, ^7^, ^8^ that after excitation the molecule can either go to the triplet manifold or to the dissociative ${S{}^{'}}_{CT}\left(\pi \pi^{*}\right)$ state, but not via IC to the ground state, then the triplet and photodegradation quantum yields should add up approximately to 1 ($\Phi_{\mathrm{trip}}+\Phi_{\deg}\approx 1$ ). This assumption is fulfilled for 2NN, where $\Phi_{\mathrm{trip}}\approx 1$ .^8^ However, for 1NN and 2M1NN, $\Phi_{\mathrm{trip}}$ and $\Phi_{\deg}$ add up to ≈64 and 45 %, respectively.^8^ Thus, 36 and 55 % of the yields in the excited‐state dynamics in 1NN and 2M1NN are not accounted for, which questions the above assumption that $S_{1}\to S_{0}$ IC does not occur in any NN derivative. In our nonadiabatic dynamics simulations, we observed some IC to the ground state, which was unfortunately not statistically significant to extract a time scale. To solve this issue, longer propagation times are necessary and foremost a method beyond the single‐reference TDDFT, that can naturally describe transitions from *S*_1_ to *S*_0_.^61^

## 6 Conclusions and Outlook

In this work, we investigated the excited‐state dynamics of three NN derivatives: 2NN, 1NN, and 2M1NN. While previous time‐resolved spectroscopic experiments^2^, ^3^, ^4^, ^5^, ^6^, ^7^, ^8^ were interpreted as to show ISC in the NN derivatives on a time scale of ∼100 fs, this work concludes that such a time constant corresponds to relaxation dynamics within the singlet manifold. Our nonadiabatic dynamics simulations based on TDDFT calculations demonstrates that after the initial ultrafast dynamics in the singlet manifold, ISC occurs on the time scale of ∼1 ps for the NN derivatives investigated. In particular we find that ISC occurs via two distinct pathways, a $S_{LE}\left(n\pi^{*}\right)\to T_{LE}\left(\pi {}^{'}\pi^{*}\right)$ and a $S_{CT}\left(\pi \pi^{*}\right)\to T_{LE}\left(n\pi^{*}\right)$ transition, both of which follow El‐Sayed's rules. The pathways play different roles in the excited‐state dynamics of the three NN derivatives. The $S_{LE}\left(n\pi^{*}\right)\to T_{LE}\left(\pi {}^{'}\pi^{*}\right)$ pathway accounts for most of the ISC in 2NN and 1NN, whereas both pathways have similar contributions to ISC in 2M1NN. The electronic transition in the $S_{LE}\left(n\pi^{*}\right)\to T_{LE}\left(\pi {}^{'}\pi^{*}\right)$ pathway is localized completely on the nitro group so it can expected to be active in a wide range of nitroaromatic molecules, as long as the initial $S_{LE}\left(n\pi^{*}\right)$ state is efficiently populated.

In light of the present results, the existence of ISC as fast as ∼100 fs in other nitroaromatic molecules should be questioned. Xu and co‐workers performed nonadiabatic dynamics simulations on nitrophenols,^33^, ^34^ predicting $S_{1}\to T_{2}$ ISC to occur within <25 fs, despite SOCs between both states were found to be ca. 40 cm^−1^, i. e., of the same size as those associated to the $S_{LE}\left(n\pi^{*}\right)$ and $T_{LE}\left(\pi \pi^{*}\right)$ states of the NN derivatives found in this work. However, their use of modified initial conditions with artificially increased kinetic energies and their use of a global switching algorithm that forces hops, when an energy functional given by the quotient of the differences of the adiabatic and corresponding diabatic potential energies of two states reaches a local maximum, is likely to artificially enhance the hopping rates. Two studies using photoelectron spectroscopy and transient absorption spectroscopy report that one of the primary relaxation pathways of 2‐nitrophenol is sub‐picosecond IC interval conversion from the *S*_1_ to the ground state, and it is only speculated that ISC to the triplet states competes at a similar time scale.^26^, ^27^ This IC to the ground state is completely missing in the simulations of Xu and co‐workers, thus, further hinting towards an artificially large ISC rate in their simulations.

In conclusion, despite small and deceptively simple, NN derivatives still leave important questions unanswered such as the role of IC to the ground state. Further simulations require longer propagation times as well as likely methods going beyond the single‐reference DFT/TDDFT description.

## Conflict of interest

The authors declare no conflict of interest.
